# Comprehensive Evaluation on Soil Properties and *Artemisia ordosica* Growth under Combined Application of Fly Ash and Polyacrylamide in North China

**DOI:** 10.3390/e22020148

**Published:** 2020-01-27

**Authors:** Jiping Niu, Xiaoling Su, Zejun Tang, Kaiwen Lu, Gengxi Zhang, Fengxin Wang, Jie Wang

**Affiliations:** 1 College of Water Resources and Architectural Engineering, Northwest A&F University, Yangling 712100, China; niujp@nwafu.edu.cn (J.N.); lukaiwen@nwafu.edu.cn (K.L.); gengxizhang@nwsuaf.edu.cn (G.Z.); 2 Key Laboratory of Agricultural Soil and Water Engineering in Arid and Semiarid Areas, Ministry of Education, Northwest A&F University, Yangling 712100, China; 3 College of Water Resources and Civil Engineering, China Agricultural University, Beijing 100083, China; tangzejun@sina.com (Z.T.); fxinwang@cau.edu.cn (F.W.); 4 Water and Soil Conservation Station of Baoding, Baoding 071000, China; wangjie@bdstbc.cn

**Keywords:** desertification control, entropy weight method, fly ash (FA), anionic polyacrylamide (PAM)

## Abstract

A field experiment was conducted to investigate the combined application effects of fly ash (FA) (0, 5%, 10%, and 15% (*w*/*w*) soil) and polyacrylamide (PAM) (0, 0.006% and 0.012% (*w*/*w*) soil) on the edge of Hobq Desert in Inner Mongolia, China from May 2016 to October 2018. Seven different ratios of FA and PAM were selected as evaluation objects, a total of 14 soil property indices and 9 *Artemisia ordosica* growth indices were selected as evaluation indicators, and the entropy weight method was employed to evaluate the soil physicochemical properties and vegetation growth performances under FA and PAM amendments. The results showed that the F15P1 (15% FA + 0.006% PAM) and F5P1 (5% FA + 0.006% PAM) were the effective treatments for soil improvement and *Artemisia ordosica* growth respectively. Considering the soil properties and *Artemisia ordosica* growth in 2016–2018 synthetically, the highest score was observed in the F5P1, followed by the F5P2 (5% FA + 0.012% PAM) and F10P1 (10% FA + 0.006% PAM) treatments. The optimal amounts for FA and PAM should be recommended as 5% and 0.006%, respectively.

## 1. Introduction

China is one of the countries that is seriously affected by desertification in the world; the desertified and sandified land area reached up to 4.33 × 10^6^ km^2^, especially in the northern arid and semi-arid region. In these regions, under intensive interference of human activities, the erosion, migration and accumulation of loose sandy sediments on the surface of the earth, which is mainly driven by wind, is more likely to occur. The desertification and sandification has led to the impoverishment of land, which is characterized by the negative changes in soil physical, chemical, and biological properties [1,2,3,4] and the loss of natural vegetation [5]. The quality of soil is the key factor to affect the survival and development of desert plants in arid environment [6], and growth situation of plants can also effectively reflect soil conditions. Therefore, the study on desertification control plays an important role in the restoration and reconstruction of desert ecosystem in arid and semi-arid areas.

Fly ash (FA) is the most abundant combustion residue of coal in thermal power plants [7] composed of porous mineral particles. Because of its unique structure and properties (such as small bulk density, large porosity, and specific surface area, good aeration, and water perviousness), FA has been widely used in environmental ecology and other fields [8]. Polyacrylamide (PAM) is a kind of high molecular compound that has a strong flocculation effect. PAM can help to bond soil particles, consolidate topsoil, optimize the permeability and fertility of soil, and promote the growth of plants [9]. Yang et al. [10] demonstrated that the combined application of FA and PAM to sandy soil increased the resistance to wind erosion significantly. Pathan et al. [11] found that the FA and PAM increased the field capacity and the content of effective soil water. Moreover, through a field experiment, Zhao et al. [12] proved that the corn yield increased in a mixture of sandy soil, 5% (*w*/*w*) FA and PAM, while decreased in a mixture of sandy soil, 10% and 15% (*w*/*w*) FA and PAM. The action of FA and PAM was related to the application amount, soil type, and environmental factors. Therefore, it is necessary to investigate the effectiveness and to select the optimum proportion of combined application of FA and PAM in soil improvement and vegetation restoration objectively and comprehensively.

The concept of entropy originated from thermodynamics; Shannon applied the concept in the field of information theory in 1948, which was called information entropy. Information entropy is a measure of the disorder degree of a system. In the entropy weight method, the natural information of proposed observation indexes are fully considered and individual subjective factors are avoided in the process of determining the weights of evaluation indicators. Ultimately the entropy weight method improve the scientificity and objectivity of the evaluation results. At present, the entropy weight method has been widely used in water quality appraisal [13,14], streamflow forecasting [15], image thresholding [16], cotton salt tolerance assessment [17] in a lot of practice. However, there is very little research that has reported the application of this method in evaluating the effect of desert vegetation restoration and environmental reconstruction under FA and PAM amendments.

In this paper, the entropy weight method was used to analyze the effects of combined application of FA and PAM, and find out the optimal application ratio, thereby to afford theoretical guide and technical support for desert vegetation restoration and ecological environment reconstruction under the background of desertification control. A field experiment was conducted from May 2016 to October 2018 in the Hobq Desert, which was characterized by dry climate, serious hazard of wind sand, and extremely fragile ecological environment in north China. The representative indexes were selected including ten soil property indexes and nine vegetation growth indexes to comprehensively and objectively investigate the effect on combined application of FA and PAM on sandy soil improvement and *Artemisia ordosica* growth. Specifically, we addressed the following questions: (1) To use entropy weight method for evaluating the effects of combined application of FA and PAM in North China; (2) to select the optimum proportion of FA and PAM for soil improvement and *Artemisia ordosica* growth; (3) to analyze the mechanism of the actions of FA and PAM in North China.

## 2. Material and Methods

### 2.1. Study Area

The study area is located on the edge of Hobq Desert (40°16′ N–40°39′ N, 107°45′ E–109°50′ E, altitude 1020–1097 m), the Hobq Desert is in Inner Mongolia, North China. It has a desert area of 13,358 km^2^, ranking as the seventh largest desert in China. The desert is in a typical semi-arid temperate continental monsoonal climate zone. According to the Chinese International Exchange Stations Surface Climate Standard Values Monthly Dataset (1971–2000) (weather station No.:53463; location: 40°49′ N, 111°41′ E, 1063 m asl) (National Meteorological Information, 2005), the mean annual temperature is 6.1 °C, and the lowest and highest monthly mean temperatures are -34.5 °C and 40.2 °C, respectively. The mean annual precipitation is 250 mm, falling predominantly during summer, and the mean annual pan evaporation is 2160 mm. The annual average sunshine hours are about 3205 h, and the frost-free period is about 125 days. The mean annual wind speed is 3.9 m/s, and from March to May each year, the wind speed can over 30 m/s. The land desertification in the study area is serious and the activities of wind and sand are strong. The dominant species of natural plant are *Artemisia ordosica*, *Agriophyllum squarrosum*, and *Elymus dahuricus*, and the main afforestation tree species are *Caragana microphylla*, *Salix mongolica*, and *Hedysarum mongolicum*.

### 2.2. Experimental Design

The field experiment was carried out from May 2016 to October 2018. There were seven treatments as shown in Table 1 (*w*/*w*), composed of three addition rates of FA (5%, 10%, and 15% (*w*/*w*) soil) and two addition rates of PAM (0.006% and 0.012% (*w*/*w*) soil). FA and PAM were evenly applied to 0–30 cm soil depth. CK treatment was a blank control group with no FA or PAM in it. Each treatment was repeated for three times.

The FA applied in the experiment was taken from Inner Mongolia Hong Zhu Thermal Power Plant, and gently crushed and sieved through the 2-mm stainless steel sieve before the experiment. The PAM used in the experiment was white powder with a relative molecular mass of more than 3 million anions and a solid content of 85% (*w*/*w*). Subsequently, the *Artemisia ordosica* was planted on sandy soil improved by FA and polyacrylamide. The *Artemisia ordosica*, a semi shrub of the genus *Artemisia* with full leaf pinnate, is widely distributed in the desert areas of North China. It is also the dominant species of natural plant communities with good sand fixing function and important ecological values in Hobq Desert.

The soil samples were collected from 0–30 cm topsoil. The particle size distribution was determined by the laser diffraction method using a particle size analyzer (Mastersizer 2000, Malvern, UK). The soil bulk density, total porosity and capillary porosity, saturated water content, field water-holding capacity, and soil void ratio were obtained by the indoor cutting-ring soaking method. The pH and electrical conductivity (EC) were measured in a soil/water mixture at the ratio of 1:5 using a pH meter and a conductivity meter respectively. Total nitrogen was determined by the Kjeldahl method, total phosphorous was determined by Mo-Sb colorimetric method. Physicochemical properties of the FA and soil used in the experiment are shown in Table 2. The seed germination of *Artemisia ordosica* were tested from 25 May 2016. Started from the appearing of the first true leaf of the seedling, the number of seedlings per plot was recorded daily, and the total percent of seedling emergence of *Artemisia ordosica* was counted for 8 days. Ten strains were selected randomly in each plot and the plant height (PLH), basal stem diameter (BD), and leaf number (LN) were measured every 15 days. Besides, five strains were selected randomly in each plot and dried in an oven of 85 °C for 48 h every 30 days to obtained the total fresh weight (TFW), total dry weight (TDW), dry weight aboveground (ADW), and dry weight underground (UDW).

The seed emergence rate is defined as:

(1) $$SER=\frac{n}{N}\times 100\%$$

where *SER* is the seed emergence rate (%); *n* is the total number of emerging seeds; and *N* is the total number of seeds.

The vigor index can be expressed as:

(2) $$VI=S\times \sum \frac{Gt}{Dt}$$

where *VI* is the seedling vigor index; S is fresh weight of seedlings (g); Gt is the number of seedlings emerged on the t day; Dt is the corresponding number of germination days.

### 2.3. Comprehensive Evaluation Based on Entropy Weight Method

Suppose there are n evaluation objects and m evaluation indexes, the original index data matrix *X* is formed as:

(3) $$X=\left[\begin{array}{cccc} X_{11} & X_{12} & \ldots & X_{1m}\\ X_{21} & X_{22} & \ldots & X_{2m}\\ \ldots & \ldots & \ldots & \ldots \\ X_{n1} & X_{n2} & \ldots & X_{nm} \end{array}\right]$$

Because of the different dimension and magnitude of the m indexes, it is necessary to standardize the m indexes to ensure the objectivity and scientificity of the analysis results. The membership function is used to deal with each index to convert it into [0, 1] to make the m indexes comparable, the standardized values are as follows:

(4) $$P_{ij}=\frac{X_{ij}-\min\left(X_{i}\right)}{\max\left(X_{i}\right)-\min\left(X_{i}\right)},\;\left(1\leq i\leq n,1\leq j\leq m\right)$$

The formulas for calculating information entropy ($E_{j}$) and entropy weight (*Wj*) of each index are obtained as follows:

(5) $$E_{j}=-\frac{1}{\ln\;n}\sum_{i=1}^{n}P_{ij}\cdot \ln\;P_{ij}\;\left(\mathrm{if}\;P_{ij}=0,\;\ln\;P_{ij}=0\right)$$

(6) $$W_{j}=\frac{1-E_{j}}{\sum_{j=1}^{m}\left(1-E_{j}\right)}$$

The comprehensive evaluation value (*Fi*) of the j index can be calculated by:

(7) $$F_{i}=\sum_{j=1}^{m}X_{ij}\cdot W_{j}$$

## 3. Results

### 3.1. Comprehensive Evaluation on Soil Properties

According to the principles of representativeness, dominance, stability, diversity, and comparability, ten soil physical and chemical indexes are selected as soil evaluation indexes, which reflect the arrangement of soil particles, soil porosity, soil nutrients, and fertility, making the comprehensive evaluation of soil improvement more scientific and reasonable. As shown in Table 3, the sample matrix *X* was composed of ten soil evaluation indexes and seven FA and PAM mixing ratios through the field experiment.

Information entropy theory can reflect the utilization degree of evaluation indexes, which means it can quantify the importance of the indexes. In a multi-index comprehensive evaluation system, the larger entropy weight (*Wj*) of an evaluation index represents smaller information entropy (*Ej*), greater variation degree; it also means more information was provided. As shown in Table 4, *Ej* and *Wj* of the ten soil indices were calculated by the entropy weight method, and the greater the dispersion of an index value, the more significant impact of this index on sandy soil improvement was. The total sequence of *Wj* was obtained, the order was pH (*X*7) > total nitrogen (*X*9) > soil capillary (*X*3) > total soil porosity (*X*2) > soil conductivity (*X*8) > soil void ratio (*X*6) > total phosphorous (*X*10) > soil saturation content (*X*4) > field water-holding capacity (*X*5) > soil bulk density (*X*1).

In order to compare the effects of soil improvement under different mixing ratios of FA and PAM more intuitively, the comprehensive score of each treatment was gained using formula 7. The results are shown in Table 5, F15P1 treatment got the highest comprehensive score, followed by F15P2 treatment, F15P1 and F15P2 treatments scored over 16.10. CK gained the lowest comprehensive score of 13.74. The results showed that the combined application of FA and PAM had significant improvement effects on soil properties, and the proportioning of FA and PAM affected the impacts.

### 3.2. Comprehensive Evaluation on Artemisia ordosica Growth

Table 6 showed the SER, VI, PLH, BD, LN, TFW, TDW, ADW, and UDW of *Artemisia ordosica* measured in 2016–2018, among them, the SER and VI represented the plant growing status in seedling stage in 2016. To assess the growth of *Artemisia ordosica* on FA and PAM mixtures in 2016, 2017, and 2018 respectively, three sample matrixes were established according to Table 6.

By comparing *Ej* and *Wj*, the index that provides more information and has greater impact on the growth of *Artemisia ordosica* can be identified in 2016–2018. As seen in Table 7, the rank of Wj in 2016 was in the order SER (*Y*1) > TFW (*Y*6) > UDW (*Y*9) > BD (*Y*4) > VI (*Y*2) > ADW (*Y*8) > TDW (*Y*7) > PLH (*Y*3) > LN (*Y*5), therefore, the SER and biomass had a great influence on evaluating the differences growth characteristics under combined application of FA and PAM. In 2017, the *Wj* value of UDW was the largest with a value of 0.172, followed by TDW, ADW, and TFW. Similarly, in 2018 the maximum *Wj* index was TFW with the value of 0.1925.

The *Artemisia ordosica* growth indicators and the calculated *Wj* values were substituted into formula 7 to obtain the comprehensive scores under different treatments. As shown in Table 8, in 2016, 2017, 2018 and 2016–2018, the order of FA and PAM treatments were basically the same. CK, F5P1, and F5P2 were always the top three optimum ratios for *Artemisia ordosica* growth, while F15P2 gained the lowest comprehensive score in different growth phases of *Artemisia ordosica* constantly. The optimum mixing ratio of FA and PAM for *Artemisia ordosica* growth in 2016–2018 has been valued synthetically. The results showed that F5P1 was the most suitable ratio, F15P2 was the most hostile mixture ratio at once (Table 9).

### 3.3. Correlations among Soil Property and Artemisia Ordosica Growth Parameters

The relationships between soil property (*X*1–*X*10) and *Artemisia ordosica* growth (*Y*1–*Y*9) parameters were listed in Table 10. There were significant correlations among soil bulk density, total porosity, capillary porosity, saturated water content, field water-holding capacity, soil void ratio, and conductivity, negative correlations were found between the soil bulk density and other soil parameters. The levels of TDW, ADW, UDW also showed significant positive correlations with SER and VI as well as TFW. In general, the soil bulk density, saturated water content, field water-holding capacity and soil void ratio were strongly correlated with the *Artemisia ordosica* growth parameters other than VI.

In this paper, seven different ratios of FA and PAM were selected as evaluation objects, a total of 14 soil property indices and 9 *Artemisia ordosica* growth indices were selected as evaluation indicators, and the entropy weight method was employed to evaluate the soil physicochemical properties and vegetation growth performances under FA and PAM amendments. According to the comprehensive three-year study, the effect of FA and PAM mixture on desertification control has been quantitatively evaluated through analyzing the comprehensive scores, the authors confirmed that the mixture ratio of F5P1 and F5P2 could improve the sandy soil properties and promote vegetation growth more effectively. The results will provide an important theoretical basis for the rational application of FA and PAM for desertification control, desert vegetation restoration and ecological reconstruction.

## 4. Discussion

In the comprehensive evaluation on the effects of combined application of FA and PAM, the entropy weight method was introduced to analyze a large number of data composed by the soil properties and *Artemisia ordosica* growth parameters. The Wj was determined according to the information each parameter carried, therefore the information stored in the data were fully utilized, the objective and reliable comprehensive scores and rankings were obtained ultimately by the entropy weight method, which overcame the lack of unified evaluation standard and reduced the interference of human subjectivity to the evaluation processes and results. At the same time, correlation analysis objectively reflected the relationships among the soil property and *Artemisia ordosica* growth parameters.

The correlation analysis in Table 10 identified that the soil physical properties and chemical properties had correlation in different degrees. The soil physical and chemical properties interacted with each other and affected the soil quality jointly. Moreover, the soil bulk density, saturated water content, field water-holding capacity, and soil void ratio were strongly correlated with the *Artemisia ordosica* growth parameters (except for VI). The growth status of *Artemisia ordosica* was a comprehensive result after FA and PAM affecting soil physicochemical environment and microbial activities. The results demonstrated that the soil properties, and the growth of *Artemisia ordosica* could be characterized by each other.

The comprehensive evaluation of soil physical and chemical properties can directly reflect the general condition of soil. As shown in Table 5, the comprehensive scores of the six mixed FA and PAM ratios were all higher than CK, this proved that the FA and PAM improved the physicochemical properties of sandy soil as well as the comprehensive soil quality. The reason for this is that the FA is a kind of inorganic material, which has the characteristics of low bulk density, large specific surface area, and good ventilation, while PAM is a kind of polymer with strong polymerization. The composite application of FA and PAM enhanced the cohesive forces among soil particles, promoted the structural stability of soil, and improved the sandy soil properties through filling small FA particles into the large soil pores as well as the flocculation effect of PAM [18]. Table 8 indicated that for *Artemisia ordosica* growth, the low FA application (F5P1 and F5P2) got higher comprehensive scores than that of high FA application (F10P1, F10P2, F15P1, and F15P2). On the one hand, FA and PAM improved soil physicochemical properties, FA also contained rich nutrient including Mg, K, Na and trace elements B, Zn, Cu at once [11]. On the other hand, the high proportion of FA had strong alkalinity and high salt content, which increased the pH and conductivity of soil, at the same time, the lack of C, N, and other elements in FA may lead to the inhibition of microbial activities, although the application of PAM alleviated the negative effects of FA to a certain extent. This finding agreed with the studies of Zhao et al. [12], who observed that the corn yield treated by 5% FA was 9% higher than that of control group, but the application of 10% and 15% FA would lead to corn yield reduction.

According to the calculation results of entropy weight method, the comprehensive scores of soil properties in F15P1, F15P2, and F5P2 were higher than the other treatments (Table 5). However, the significant growth of *Artemisia ordosica* in F5P1 and F5P2 during 2016–2018 indicated that the addition of FA at a rate of 5% proved to be the most effective ratio to promote vegetation growth. It can be discovered that when FA and PAM were mixed with sandy soil, the same proportion had different effects on soil improvement and vegetation growth. Therefore, the soil property and *Artemisia ordosica* growth indicators in 2016–2018 were adopted to calculate comprehensive scores for selecting the optimal treatment. The results are shown in Table 9, F5P1, CK, and F5P2 were prominently the top three score treatment.

The results of entropy weight method reflected the soil quality and *Artemisia ordosica* growth situation actually under different FA and PAM proportions, which was of great significance for the rational application of FA and PAM in desert areas and the desertification control. According to the comprehensive three-year study, the authors confirmed that the mixture ratio of F5P1 and F5P2 can improve the sandy soil properties and promote vegetation growth more effectively. However, with the weathering of FA, the leaching of precipitation and the continuous growth of *Artemisia ordosica* root system, the positive effect of FA on soil improvement and desert plant growth would gradually occupy a dominant position. At present, there are few researches on the comprehensive evaluation of soil properties and *Artemisia ordosica* growth under combined application of FA and PAM, therefore, future investigation and verification are needed.

## 5. Conclusions

In this paper, FA and PAM were applied in sandy soil in a field experiment in 2016–2018. Based on the soil property and *Artemisia ordosica* growth data, the optimum mixing ratio were recommended using entropy weight method.

Different proportions of FA and PAM had various functions on the soil improvement and vegetation growth, the entropy weight method was introduced, which evaluated the efficiency of combined application results clearly by determining the weight and calculating the comprehensive score. It provided a theoretical reference for the assessment of desertification control effect in the future.

F15P1 and F15P2 improved the sandy soil physical and chemical properties effectively. While for the growth of *Artemisia ordosica*, F5P1 was the most suitable ratio, followed by F5P2, F15P2 was the most hostile treatment at once. Considering the soil properties and *Artemisia ordosica* growth in 2016–2018 synthetically, the effect of FA and PAM mixtures listed in the order F5P1 > F5P2 > F10P1 > F10P2 > F15P1 > F15P2. The result had guiding significance for the scientific management of using FA and PAM on vegetation restoration and environmental reconstruction in desert areas.

This paper only used the entropy weight method to evaluate the effects of FA and PAM mixtures on soil properties and *Artemisia ordosica* growth in 2016–2018 in North China, future investigation is needed with increasing sand-fixing desert plants types and prolonging growth time under mixed FA and PAM condition.

## Figures and Tables

**Table 1:** Design of experiment.

Treatment	FA (%, the Weight Ratio to Soil)	PAM (%, the Weight Ratio to Soil)
CK	0	0
F5P1	5	0.006
F5P2	5	0.012
F10P1	10	0.006
F10P2	10	0.012
F15P1	15	0.006
F15P2	15	0.012

**Table 2:** Physicochemical properties of the fly ash (FA) and soil used in the experiment.

Properties	Element	FA	Soil
*Physical properties*	Bulk density (g/cm^3^)	1.07	1.52
	Sand (20–2000 μm) (%)	27.4	97.9
	Silt (2–20 μm) (%)	70.8	2.03
	Clay (0.01–2 μm) (%)	1.8	0.07
	Texture	Silt loam	Sandy loam
*Chemical properties*	pH	8.42	7.25
	EC (ms/cm)	1.62	0.31
	Total nitrogen (g/kg)	-	0.15
	Total phosphorous (g/kg)	0.22	0.18

**Table 3:** Mean values of soil property indices.

Treatments	*X*1	*X*2	*X*3	*X*4	*X*5	*X*6	*X*7	*X*8	*X*9	*X*10
CK	1.53	38.25	37.20	24.99	24.31	0.62	7.25	0.28	0.15	0.18
F5P1	1.42	38.89	38.34	27.48	27.09	0.64	7.68	0.32	0.12	0.17
F5P2	1.42	40.37	39.36	28.40	27.69	0.68	7.64	0.31	0.13	0.18
F10P1	1.41	38.97	37.48	27.93	26.84	0.65	7.73	0.34	0.25	0.18
F10P2	1.40	39.53	38.29	28.27	27.38	0.66	7.71	0.35	0.25	0.18
F15P1	1.35	43.24	42.44	32.08	31.48	0.76	7.79	0.39	0.16	0.18
F15P2	1.34	42.94	42.17	32.02	31.44	0.75	7.87	0.38	0.28	0.19

Note: *X*1–*X*10: soil bulk density (g/cm^3^), total soil porosity (%), soil capillary porosity (%), saturated water content (%), field water-holding capacity (%), soil void ratio (%), pH, soil conductivity (dS/m), total nitrogen (g/kg), total phosphorous (g/kg).

**Table 4:** *Ej* and *Wj* of soil property indices.

Soil Property Indices	*Ej*	*Wj*
*X*1	0.8125	0.0609
*X*2	0.675	0.1055
*X*3	0.6333	0.1191
*X*4	0.7447	0.0829
*X*5	0.7499	0.0812
*X*6	0.7054	0.0957
*X*7	0.5708	0.1394
*X*8	0.6956	0.0988
*X*9	0.6018	0.1293
*X*10	0.7312	0.0873

Note: *X*1–*X*10: soil bulk density (g/cm^3^), total soil porosity (%), soil capillary porosity (%), saturated water content (%), field water-holding capacity (%), soil void ratio (%), pH, soil conductivity (dS/m), total nitrogen (g/kg), total phosphorous (g/kg).

**Table 5:** Comprehensive scores and rankings of soil property under different treatments.

	CK	F5P1	F5P2	F10P1	F10P2	F15P1	F15P2
Fi	13.74	14.43	14.83	14.38	14.60	16.15	16.10
rank	7	5	3	6	4	1	2

**Table 6:** Mean values of *Artemisia ordosica* growth indices in 2016–2018.

	Treatments	*Y*1	*Y*2	*Y*3	*Y*4	*Y*5	*Y*6	*Y*7	*Y*8	*Y*9
2016	CK	44.10	7.64	114.73	14.50	1291	173.99	74.94	64.81	10.13
	F5P1	41.54	9.08	88.95	12.15	938	181.42	56.77	46.07	10.70
	F5P2	44.23	8.36	82.50	11.29	745	94.46	37.24	29.70	7.54
	F10P1	40.64	4.77	85.75	13.67	482	60.80	26.91	22.57	4.34
	F10P2	40.96	4.64	88.38	13.24	467	49.94	24.33	20.45	3.88
	F15P1	35.26	3.14	75.00	10.03	318	26.85	12.84	10.99	1.85
	F15P2	35.90	4.08	74.75	7.99	257	28.50	14.13	11.63	2.50
2017	CK			167.00	17.42	9210	619.52	260.19	226.57	33.62
	F5P1			166.50	16.69	13930	658.69	279.19	241.82	37.37
	F5P2			145.00	15.58	7932	220.94	85.92	80.69	5.23
	F10P1			129.50	13.69	6184	264.67	116.42	104.62	11.80
	F10P2			116.00	13.28	5224	80.09	37.84	34.07	3.77
	F15P1			122.00	10.37	3030	200.12	98.97	87.50	11.47
	F15P2			107.00	8.87	2262	66.62	37.83	31.96	5.87
2018	CK			168.50	19.27	16231	774.98	314.00	272.52	41.48
	F5P1			170.75	19.32	18087	771.37	323.88	279.02	44.86
	F5P2			150.50	17.59	7161	295.32	178.94	146.77	32.18
	F10P1			136.75	14.49	6850	283.18	170.65	148.58	22.07
	F10P2			127.00	14.22	5113	257.11	201.34	177.34	24.01
	F15P1			127.50	11.64	5239	270.10	163.73	142.65	21.09
	F15P2			120.25	11.17	3762	210.65	135.79	122.42	13.37

Note: *Y*1–*Y*9: SER (%), VI; PLH (cm), BD (mm), LN, TFW (g), TDW (g), ADW (g), UDW (g).

**Table 7:** Ej and Wj of *Artemisia ordosica* growth indices in 2016–2018.

Year		*Y*1	*Y*2	*Y*3	*Y*4	*Y*5	*Y*6	*Y*7	*Y*8	*Y*9
2016	Ej	0.538	0.6778	0.7397	0.662	0.7458	0.5518	0.6891	0.6861	0.6338
	Wj	0.1502	0.1048	0.0846	0.1099	0.0827	0.1457	0.1011	0.102	0.1191
2017	Ej			0.6663	0.6374	0.7981	0.618	0.5707	0.6035	0.5624
	Wj			0.1312	0.1426	0.0794	0.1502	0.1688	0.1559	0.172
2018	Ej			0.6494	0.5623	0.6423	0.5125	0.6948	0.6479	0.7584
	Wj			0.1385	0.1728	0.1413	0.1925	0.1205	0.139	0.0954

Note: *Y*1–*Y*9: SER (%), VI; PLH (cm), BD (mm), LN, TFW (g), TDW (g), ADW (g), UDW (g).

**Table 8:** Comprehensive scores and rankings of *Artemisia ordosica* growth under different treatments in 2016–2018.

Year		CK	F5P1	F5P2	F10P1	F10P2	F15P1	F15P2
2016	Fi	166.19	131.68	98.77	69.56	66.45	45.87	41.33
	rank	1	2	3	4	5	6	7
2017	Fi	933.37	1319.93	711.88	587.42	456.08	320.36	217.17
	rank	2	1	3	4	5	6	7
2018	Fi	2548.12	2812.36	1137.19	1086.86	842.96	853.20	625.14
	rank	2	1	3	4	6	5	7
2016–2018	Fi	1145.51	1335.18	612.66	547.16	429.24	382.31	277.56
	rank	2	1	3	4	5	6	7

**Table 9:** Comprehensive scores and rankings of soil property and *Artemisia ordosica* growth in 2016–2018 under different treatments.

	CK	F5P1	F5P2	F10P1	F10P2	F15P1	F15P2
Fi	835.18	973.04	448.73	401.08	315.55	281.91	205.87
rank	2	1	3	4	5	6	7

**Table 10:** Correlation coefficient matrix of soil property and *Artemisia ordosica* growth parameters.

	*X*1	*X*2	*X*3	*X*4	*X*5	*X*6	*X*7	*X*8	*X*9	*X*10	*Y*1	*Y*2	*Y*3	*Y*4	*Y*5	*Y*6	*Y*7	*Y*8	*Y*9
*X*1	1	−0.818 *	−0.798 *	−0.942 **	−0.930 **	−0.841 *	−0.974 **	−0.937 **	−0.454	−0.371	0.833 *	0.653	0.967 **	0.804 *	0.938 **	0.801 *	0.923 **	0.936 **	0.797 *
*X*2		1	0.991 **	0.962 **	0.967 **	0.998 **	0.675	0.841 *	0.251	0.583	−0.852 *	−0.649	−0.782 *	−0.912 **	−0.751	−0.736	−0.771 *	−0.766 *	−0.75
*X*3			1	0.945 **	0.962 **	0.983 **	0.652	0.807 *	0.166	0.514	−0.838 *	−0.566	−0.761 *	−0.936 **	−0.687	−0.646	−0.702	−0.701	−0.665
*X*4				1	0.996 **	0.973 **	0.848 *	0.936 **	0.374	0.517	−0.901 **	−0.703	−0.901 **	−0.898 **	−0.880 **	−0.807 *	−0.882 **	−0.884 **	−0.819 *
*X*5					1	0.974 **	0.830 *	0.918 **	0.319	0.485	−0.898 **	−0.656	−0.889 **	−0.921 **	−0.841 *	−0.755 *	−0.841 *	−0.845 *	−0.771 *
*X*6						1	0.705	0.865 *	0.282	0.583	−0.866 *	−0.68	−0.806 *	−0.903 **	−0.785 *	−0.767 *	−0.804 *	−0.799 *	−0.781 *
*X*7							1	0.868 *	0.486	0.276	−0.736	−0.567	−0.958 **	−0.709	−0.914 **	−0.741	−0.888 **	−0.907 **	−0.725
*X*8								1	0.538	0.445	−0.948 **	−0.849 *	−0.840 *	−0.717	−0.937 **	−0.854 *	−0.920 **	−0.913 **	−0.900 **
*X*9									1	0.698	−0.465	−0.714	−0.32	−0.215	−0.663	−0.686	−0.612	−0.589	−0.7
*X*10										1	−0.455	−0.616	−0.304	−0.529	−0.529	−0.675	−0.535	−0.506	−0.656
*Y*1											1	0.813 *	0.709	0.738	0.802 *	0.716	0.772 *	0.758 *	0.801 *
*Y*2												1	0.519	0.39	0.826 *	0.890 **	0.821 *	0.788 *	0.953 **
*Y*3													1	0.792 *	0.887 **	0.753	0.890 **	0.912 **	0.715
*Y*4														1	0.651	0.552	0.646	0.658	0.541
*Y*5															1	0.946 **	0.993 **	0.991 **	0.940 **
*Y*6																1	0.964 **	0.950 **	0.984 **
*Y*7																	1	0.998 **	0.948 **
*Y*8																		1	0.927 **
*Y*9																			1

Note: *X*1–*X*10: soil bulk density (g/cm^3^), total soil porosity (%), soil capillary porosity (%), saturated water content (%), field water-holding capacity (%), soil void ratio (%), pH, soil conductivity (dS/m), total nitrogen (g/kg), total phosphorous (g/kg). *Y*1–*Y*9: SER (%), VI; PLH (cm), BD (mm), LN, TFW (g), TDW (g), ADW (g), UDW (g). * and ** indicate significance at *p* < 0.05 and *p* < 0.01 levels, respectively.
